# The Ohio Medical and Surgical Journal

**Published:** 1850-06

**Authors:** 


*The Ohio Medical and Surgical Journal*, complains that the *Western Journal* is not received in exchange. Why this is so, we cannot tell, for our Journal is regularly mailed for the Ohio Medical and Surgical Journal. The arrangements respecting the transmission of medical periodicals are as bad as they can well be. We often fail to receive at least one half of our exchanges, but we know of no way of remedying the evil.

